# Bacterial RNA Contributes to the Down-Modulation of MHC-II Expression on Monocytes/Macrophages Diminishing CD4^+^ T Cell Responses

**DOI:** 10.3389/fimmu.2019.02181

**Published:** 2019-09-13

**Authors:** M. Ayelén Milillo, Aldana Trotta, Agustina Serafino, José Luis Marin Franco, Fábio V. Marinho, Julieta Alcain, Melanie Genoula, Luciana Balboa, Sergio Costa Oliveira, Guillermo H. Giambartolomei, Paula Barrionuevo

**Affiliations:** ^1^Instituto de Medicina Experimental (CONICET-Academia Nacional de Medicina), Buenos Aires, Argentina; ^2^Departamento de Bioquímica e Imunologia, Universidade Federal de Minas Gerais, Belo Horizonte, Brazil; ^3^Instituto de Inmunología, Genética y Metabolismo, Hospital de Clínicas “José de San Martín” (CONICET-UBA), Buenos Aires, Argentina

**Keywords:** *Brucella abortus*, bacterial RNA, monocytes/macrophages, MHC, antigen presentation/processing

## Abstract

*Brucella abortus*, the causative agent of brucellosis, displays many resources to evade T cell responses conducive to persist inside the host. Our laboratory has previously showed that infection of human monocytes with *B. abortus* down-modulates the IFN-γ-induced MHC-II expression. *Brucella* outer membrane lipoproteins are structural components involved in this phenomenon. Moreover, IL-6 is the soluble factor that mediated MHC-II down-regulation. Yet, the MHC-II down-regulation exerted by lipoproteins was less marked than the one observed as consequence of infection. This led us to postulate that there should be other components associated with viable bacteria that may act together with lipoproteins in order to diminish MHC-II. Our group has recently demonstrated that *B. abortus* RNA (PAMP related to pathogens' viability or *vita*-PAMP) is involved in MHC-I down-regulation. Therefore, in this study we investigated if *B. abortus* RNA could be contributing to the down-regulation of MHC-II. This PAMP significantly down-modulated the IFN-γ-induced MHC-II surface expression on THP-1 cells as well as in primary human monocytes and murine bone marrow macrophages. The expression of other molecules up-regulated by IFN-γ (such as co-stimulatory molecules) was stimulated on monocytes treated with *B. abortus* RNA. This result shows that this PAMP does not alter all IFN-γ-induced molecules globally. We also showed that other bacterial and parasitic RNAs caused MHC-II surface expression down-modulation indicating that this phenomenon is not restricted to *B. abortus*. Moreover, completely degraded RNA was also able to reproduce the phenomenon. MHC-II down-regulation on monocytes treated with RNA and L-Omp19 (a prototypical lipoprotein of *B. abortus*) was more pronounced than in monocytes stimulated with both components separately. We also demonstrated that *B. abortus* RNA along with its lipoproteins decrease MHC-II surface expression predominantly by a mechanism of inhibition of MHC-II expression. Regarding the signaling pathway, we demonstrated that IL-6 is a soluble factor implicated in *B. abortus* RNA and lipoproteins-triggered MHC-II surface down-regulation. Finally, CD4^+^ T cells functionality was affected as macrophages treated with these components showed lower antigen presentation capacity. Therefore, *B. abortus* RNA and lipoproteins are two PAMPs that contribute to MHC-II down-regulation on monocytes/macrophages diminishing CD4^+^ T cell responses.

## Introduction

For several years, the research in brucellosis was focused on understanding how *B. abortus* establishes a persistent infection inside its intracellular niche, the macrophage (1–5). Once inside the macrophage, *B. abortus* traffic through early and late endo/lysosomal compartments where a large percentage of bacteria are promptly eliminated (1, 2). But then, *Brucella* is able to form vacuoles derived from endoplasmic reticulum (ER) where the surviving bacteria begin to replicate dramatically (1, 3, 4). This particular ability of *Brucella* has been considered for years as the key mechanism to evade the immune response and establish a chronic infection. However, is *Brucella* really hidden from adaptive immunity? While *Brucella* is establishing its replicative niche, macrophages are able to present *Brucella*-derived peptides on MHC class I and class II molecules to T lymphocytes. Ratifying this phenomenon, CD4^+^ and CD8^+^ T cells have been found against *Brucella* in mouse, cattle, and human infections (6–9). Thus, a relevant question is how *B. abortus* persists in the presence of robust CD4^+^ and CD8^+^ T cell responses. Previous results from our laboratory demonstrated that *B. abortus* infection inhibits the IFN-γ-induced surface expression of MHC-II and MHC-I molecules on human monocytes/macrophages (10, 11). Consequently, macrophages infected with *B. abortus* exhibit decreased ability to present antigens to CD4^+^ and CD8^+^ T cells, respectively (10–12).

Regarding the MHC-II surface inhibition mediated by *B. abortus*, this phenomenon was also mimicked by HKBA (heat-killed *B. abortus*), suggesting the participation of a *Brucella* structural component (10). In line with this, a prototypical *B. abortus* lipoprotein [outer membrane protein 19 (Omp19)], decreased the surface expression of MHC-II molecules (10). Furthermore, all *Brucella* lipoproteins are capable of inhibiting MHC-II surface expression since Pam_3_Cys (a synthetic lipohexapeptide that resemble the protein lipid moiety structure) also inhibited MHC-II expression (10). On the other hand, TLR (Toll-like receptor) 2 was the receptor involved in the MHC-II down-regulation mediated by HKBA or L-Omp19 (lipidated Omp19), and IL-6 was a soluble mediator implicated in this phenomenon (10). Recently we demonstrated that *B. abortus* lipoproteins inhibit MHC-II surface expression by decreasing the transcription of MHC-II genes (13). More specifically, *B. abortus* lipoproteins via IL-6 secretion inhibit the expression and activation of IFN-γ-induced IRF-1, decreasing the transcription of CIITA (the MHC-II master regulator) (13).

Despite the advances in the knowledge of MHC-II down-modulation by *B. abortus*, what specially caught our attention was that HKBA or *B. abortus* lipoproteins were less efficient at reducing IFN-γ-induced MHC-II surface expression than live bacteria (10, 13). Therefore, another component related to bacterial viability must be implicated in the down-modulation of MHC-II molecules. Recently, we have elucidated that the component of *B. abortus* responsible for the diminished MHC-I surface expression is its RNA, a pathogen-associated molecular pattern (PAMP) related to pathogens' viability or *vita*-PAMP (14). Interestingly, RNase-treated *B. abortus* RNA was also capable of inhibiting MHC-I expression to the same degree as native RNA (14). Both the intact molecules as well as the digested products of *B. abortus* RNA inhibit MHC-I surface expression by retaining these molecules within the Golgi apparatus (14). In addition, we demonstrated that *B. abortus* RNA down-modulates the IFN-γ-induced surface expression of MHC-I via TLR8 and by the EGFR signaling pathway (14, 15).

RNA, unlike conventional PAMPs (i.e., lipopolysaccharide, DNA and lipoproteins, among others), is found in viable bacteria but not in dead bacteria (14, 16, 17). Therefore, RNA could be another component involved in the MHC-II down-modulation in the context of *B. abortus* infection. The aim of this study was to investigate whether *B. abortus* RNA is able to modulate the IFN-γ-induced expression of MHC-II on monocytes/macrophages. Once this phenomenon was corroborated, we investigated the mechanisms and soluble mediators whereby *B. abortus* RNA alone or in combination with its lipoproteins was able to generate the inhibition of MHC-II surface expression. Finally, we evaluated if MHC-II down-modulation had biological relevance as we analyzed the antigen presentation of *B. abortus* RNA and lipoproteins-treated macrophages to CD4^+^ T cells.

## Materials and Methods

### Ethics Statement

In this study, human monocytes from adult blood donors in healthy state were utilized in agreement with the guidelines of the Ethical Committee of IMEX. Donors gave their informed consent before the study. With regard to animals, female mice from C57BL/6 strain were maintained under SPF conditions as previously described (14). All animal procedures were executed according to the rules for the use of laboratory animals of the National Institutes of Health and were authorized by the Animal Care and Use Committee of IMEX.

### Bacteria Strains and *Trypanosoma cruzi*

*B. abortus* S2308, *Escherichia coli* 11105, *Staphylococcus aureus* 25923, and *Klebsiella pneumoniae* 700603 strains were cultivated in tryptose-soy agar in which yeast extract was added (Merck). The amount of bacteria on stationary-phase cultures was determined by the comparison of the OD at 600 nm with a standard curve. The experiments that involved infection of cells with live *Brucella abortus* were performed in biosafety level 3 (BSL-3) facilities, located at the ANLIS-Malbrán (Administración Nacional de Laboratorios e Institutos de Salud, Dr. Carlos G. Malbrán) (Buenos Aires, Argentina). With regard to *T. cruzi*, trypomastigotes from the Brazil strain were cultured overnight in Dulbecco's modified Eagle medium (Mediatech; pH 5.0), to transform trypomastigotes to amastigotes, as previously described (18).

### Expression and Purification of *B. abortus* Recombinant Lipidated Omp19 (L-Omp19)

Lipoproteins were expressed in *E. coli* BL21 and purified as it was previously described (19). The final preparations contained <0.25 LPS U/μg of protein, determined by Limulus Amebocyte Lysate assay (Lonza). The purified proteins were kept at −80°C until use.

### Cell Cultures

All the experiments were carried out in an incubator at 37°C and in an atmosphere with 5% CO_2._ The standard medium used was composed of RPMI 1640 supplemented with 25 mM Hepes, 2 mM L-glutamine, 10% heat-inactivated fetal bovine serum (Gibco), 100 U of penicillin.ml^−1^ and 100 μg of streptomycin.ml^−1^. Cells from the monocytic line THP-1, were purchased from the American Type Culture Collection (ATCC, Manassas, VA) and cultured as it was formerly described (19). In order to induce differentiation to monocytes, THP-1 cells at 5 × 10^5^.ml^−1^ were cultured in 0.05 μM 1,25-dihydroxyvitamin D3 (EMD Millipore) for 72 h. In the experiments with primary human monocytes, peripheral blood mononuclear cells (PBMCs) were isolated by Ficoll-Hypaque gradient (GE Healthcare) centrifugation. Monocytes were purified from PBMCs by Percoll gradient (GE Healthcare) as previously described (10). To induce monocyte-derived DCs, monocytes were cultured at 2 × 10^6^ cell/ml under a humidified atmosphere of 5% CO_2_ at 37°C in standard medium supplemented with 50 ng/ml recombinant granulocyte-monocyte colony stimulating factor (GM-CSF) (Peprotech) and 10 ng/ml recombinant IL-4 (Prepotech) as described elsewhere (20). Bone marrow progenitors from C57BL/6 female mice were differentiated to mouse bone marrow-derived macrophages (BMDM) with recombinant monocyte colony stimulating factor (M-CSF) (PeproTech). These cells were then cultured as previously described (21).

### Viability Assay

In order to determine cellular apoptosis, 5 × 10^5^.ml^−1^ THP-1 cells were treated with *B. abortus* RNA, RNase-I-treated *B. abortus* RNA, L-Omp19 or their combination plus IFN-γ (Endogen) for 48 h. Cells treated with 2% paraformaldehyde (PFA) were included as a positive control of the technique. After 48 h, cells were washed and stained with 7-Amino-Actinomycin D (7-AAD; BD Biosciences) for 10 min at 0°C in darkness. Immediately after, cells were analyzed on a FACSCalibur® flow cytometer (BD Biosciences) or Sysmex Partec Cytometer (Sysmex Partec GmbH, Germany) and data were processed using FlowJo® 7.6 software.

### RNA Preparation

5 × 10^8^ CFU, 1 × 10^7^ amastigotes or 5–10 × 10^6^ PBMCs were suspended in 1 ml of Trizol Reagent (Invitrogen) and total RNA was isolated with *Quick*-RNA™ MiniPrep (Zymo Research) according to the manufacturer's instructions. RNA was quantified with OD at 260. The purity of the preparation was determined using a DeNovix DS-11 Spectrophotometer (DeNovix Inc.) with a ratio of absorbance 260/280 >2.0 and a ratio of absorbance 260/230 > 1.8. To exclude the potential effect of *B. abortus* DNA, some RNA preparations were incubated with DNase I (1 U/μg of RNA; Promega Corporation) in a buffer containing 400 mM Tris-HCl (pH 8.0), 100 mM MgSO_4_, and 10 mM CaCl_2_ for 30 min at 37°C and the reaction was stopped by addition of 20 mM EGTA and incubation for 10 min at 65°C. Additionally, to rule out the effect of *B. abortus* proteins in the effects mediated by *B. abortus* RNA, our preparations were treated with Proteinase K (200 μg/mL, Promega Corporation) for 60 min at 37°C, and the digestion was stopped by incubation at 96°C for 10 min.

### *In vitro* Stimulation

Cells at 5 × 10^5^.ml^−1^ were treated with *B. abortus* RNA, other prokaryotic or eukaryotic RNAs, *E. coli* RNase I (Life Technologies)-treated *B. abortus* RNA or *B. abortus* lipoproteins in the presence of 150 U.ml^−1^ IFN-γ for 48 h as it was formerly described (14). MHC-II, MHC-I, CD40, CD86, or CD80 expressions were assessed by flow cytometry. In the experiments that involved murine macrophages, BMDM were treated with different doses of *B. abortus* RNA in presence of 10 ng.ml^−1^ recombinant murine IFN-γ (PeproTech) for 48 h. Murine MHC-II expression was assessed by flow cytometry.

### Cells Infection With *B. abortus*

5 × 10^5^.ml^−1^ THP-1 were infected with a multiplicity of infection (MOI) of 100 *B. abortus* S2308 per cell in round-bottom polypropylene tubes (Falcon). This procedure was performed with 150 U.ml^−1^ IFN-γ for 2 h in standard medium without antibiotics. Afterwards, cells were extensively washed in order to remove uninternalized bacteria. Infected cells were maintained in culture in presence of IFN-γ, 100 μg.ml^−1^ gentamicin, and 50 μg.ml^−1^ streptomycin for other 48 h.

### Influence of IL-6 on MHC-II Expression

In another set of experiments, 5 × 10^5^.ml^−1^ THP-1 were incubated in presence of 150 U.ml^−1^ IFN-γ, *B. abortus* RNA (10 μg.ml^−1^) and *B. abortus* L-Omp19 (1 μg.ml^−1^) in the presence of neutralizing mAb for IL-6 (clone MQ2-13A5; eBioscience) or the isotype control at a concentration of 20 ng.ml^−1^. After this, MHC-II expression was assessed by flow cytometry.

### Flow Cytometry

Once *B. abortus* infection or stimulation of cells were performed, monocytes were stained with PE-labeled anti-human HLA-DR (clone L243, BD Pharmingen), FITC-labeled anti-human HLA-ABC (clone G46-2.6; BD Pharmingen) or isotype-matched control mAbs. To evaluate MHC-II expression, MHC-II bar graphs were performed on the MHC-II positive cells. In order to evaluate murine MHC-II surface expression, BMDMs were stained with PE-labeled anti-mouse MHC-II (I-A/I-E) (clone M5/114.15.2; e-Bioscience). To determine CD40, CD86, and CD80 surface expressions, in another set of experiments cells were stained with PE-labeled anti-human CD40 (clone 5C3; Biolegend), PE-labeled anti-human CD86 (clone IT2.2; BD) or FITC-labeled anti-human CD80 (clone 2D10; Biolegend). In all cases, monocytes were stained with 7-AAD for 10 min at 0°C in darkness. After that, all markers studied were analyzed on a FACSCalibur® flow cytometer (BD Biosciences) or Sysmex Partec Cytometer (Sysmex Partec GmbH, Germany), gating on viable cells (7-AAD negative cells). Data were processed using FlowJo® 7.6 software.

### Confocal Microscopy Experiments

Confocal micrographs were performed as previously described (11). Briefly, 2 × 10^5^ THP-1 cells/well were incubated in chamber-slides (Nunc) with 10 ng/ml PMA (Sigma-Aldrich) for 24 h to promote adherence. Then, cells were stimulated with *B. abortus* RNA (10 μg.ml^−1^), RNase I-treated *B. abortus* RNA, *B. abortus* L-Omp19 (1 μg.ml^−1^) or their combination with IFN-γ for 48 h. Afterwards, cells were treated with 2% PFA, permeabilized with 0.1% saponin and incubated with anti-HLA-DR mAb L243 (purified from murine hybridoma culture supernatants) and Alexa 546-labeled secondary Ab (Invitrogen). In order to detect Golgi apparatus, cells were labeled with a mAb specific for GM130 (BD Biosciences) followed by Alexa 488-labeled secondary Ab (Invitrogen). Then, slides were mounted and analyzed with a FV-1000 confocal microscope, as it was previously described (14).

### Ag Presentation Assay

MHC class II-restricted response to OVA was assessed using the T cell hybridoma BO97.10, specific for OVA323-339 peptide on I-Ab on macrophages. BMDMs were stimulated with the different components in presence of murine IFN-γ for 48 h. Afterwards, these cells were exposed to OVA (100 μg/ml) for 3 h at 37°C. After washing, cells were suspended in complete medium at pH 7.3 and BO97.10 cells were added for 20 h. The production of IL-2 by BO97.10 was measured by ELISA (RD) as described previously (22).

### Reagents

Neutralizing monoclonal antibody for human IL-6 (clone MQ2-13A5) was acquired from eBioscience.

### Measurement of IL-6 Secretion

Human IL-6 was measured in culture supernatants by sandwich ELISA, as it was formerly described (19).

### Statistical Analysis

Results were analyzed with one-way ANOVA followed by *post hoc* Tukey test or two-way ANOVA followed by *post hoc* Bonferroni test with GraphPad Prism software.

## Results

### *B. abortus* RNA Participates in the Down-Modulation of IFN-γ-Induced MHC-II Surface Expression by Preventing the Up-Regulation of These Molecules

**Figure 1:**
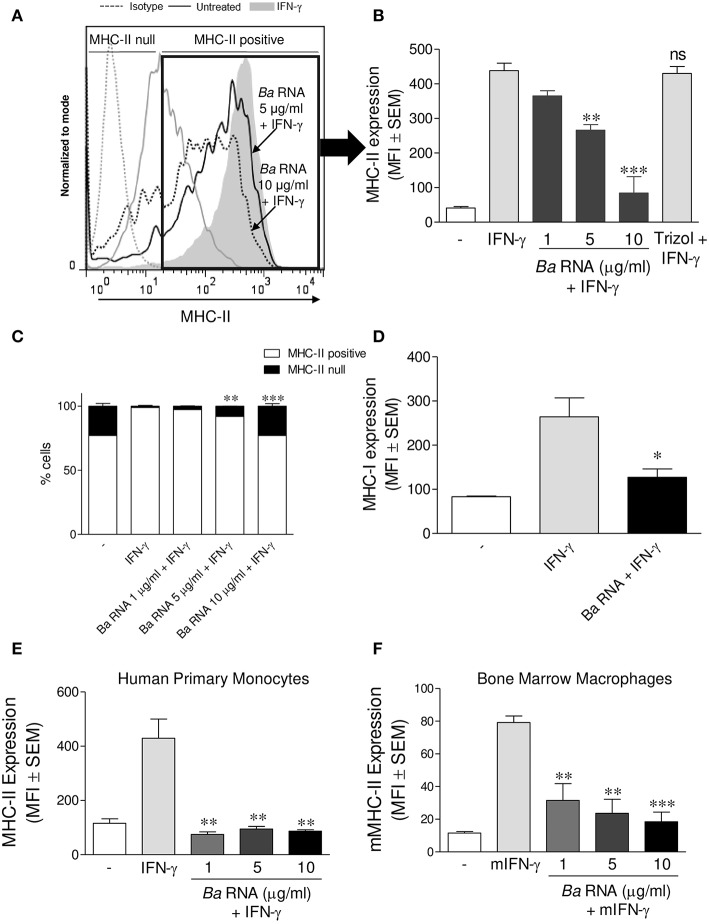
*B. abortus* RNA down-modulates MHC-II on monocytes/macrophages. **(A,B)** THP-1 cells were treated with different doses of *B. abortus* RNA in the presence of IFN-γ for 48 h. MHC-II expression was evaluated by flow cytometry. **(A)** Flow cytometry histograms (showing MHC-II positive and null cells) representative of bars showed in **(B)**. **(B)** Bars represent the arithmetic means ± SEM of MHC-II positive cells corresponding to five independent experiments. Trizol extracted products in the absence of bacteria were used as a control. **(C)** Quantification of cells expressing MHC-II (MHC-II positive cells) or not (MHC-II null). Data is expressed as the percentage of cells ± SEM of three independent experiments. **(D)** THP-1 cells were treated with *B. abortus* RNA (10 μg/ml) in the presence of IFN-γ for 48 h. MHC-I expression was evaluated by flow cytometry. **(E,F)** Peripheral blood-purified monocytes **(E)** and bone marrow macrophages **(F)** were stimulated with different doses of *B. abortus* RNA in the presence of IFN-γ for 48 h. MHC-II expression was assessed by flow cytometry. Bars represent the arithmetic means ± SEM of five independent experiments. MFI, mean fluorescence intensity; mIFN-γ, murine IFN-γ. ns, non-significant. ^*^P < 0.05; ^**^P < 0.01*;* ^***^P < 0.001 vs. IFN-γ-treated cells.

**Figure 2:**
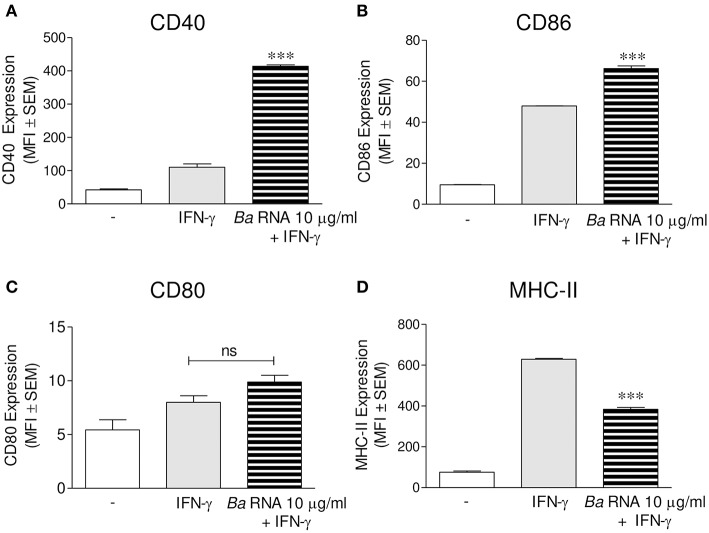
*B. abortus* RNA does not down-modulate co-stimulatory molecules. **(A–D)** THP-1 cells were treated with *B. abortus* RNA (10 μg/ml) in the presence of IFN-γ for 48 h. CD40 **(A)**, CD86 **(B)**, and CD80 **(C)** expressions were assessed by flow cytometry. MHC-II expression was determined as a control **(D)**. Bars represent the arithmetic means ± SEM of five independent experiments. MFI, mean fluorescence intensity; ns, non-significant. ^***^P < 0.001 vs. IFN-γ-treated cells.

We evaluated if *B. abortus* RNA was a *vita*-PAMP involved in MHC-II down-regulation. For this purpose, cells from the human monocytic cell line THP-1 were stimulated with *B. abortus* RNA at several doses and in presence of human IFN-γ for 48 h. Then, the surface expression of MHC-II molecules was assessed by flow cytometry. This PAMP was able to diminish the IFN-γ-up-regulated MHC-II surface expression (Figures 1A,B), as it was previously seen in *B. abortus*-infected monocytes (10, 13). Moreover, we distinguished cells expressing MHC-II (MHC-II positive cells) or not (MHC-II null cells) (Figure 1A). We observed that *B. abortus* RNA decreases the MFI of MHC-II molecules within the MHC-II-expressing population (Figures 1A,B) and slightly increases the non-expressing population in a dose-dependent manner (Figure 1C). Since some phenol remains could still appear in RNA preparations, we performed a mock RNA extraction (i.e., in the absence of bacteria), and used it as a control. MHC-II expression was not altered due to the Trizol products (Figure 1A). We also performed experiments digesting our RNA preparations with DNase and Proteinase K. Then, we evaluated the effect of these preparations on MHC-II expression. Our results demonstrated that the preparations of DNase- and Proteinase K-digested RNA were able to inhibit MHC-II expression in the same way as intact RNA, indicating that potential contaminating DNA and proteins do not mediate the phenomenon of MHC-II inhibition (Figures S1A,B in Supplementary Material). In accordance with our published results (14), MHC-I was down-modulated by *B. abortus* RNA as well (Figure 1D). In order to rule out that MHC-II down-modulation was not due to cell apoptosis, we performed all the experiments gating on viable cells (7-AAD negative cells). Moreover, we confirmed that *B. abortus* RNA treatment did not change the percentage of viable cells (Figure S2). On the contrary, when cells were treated with paraformaldehyde (PFA), high percentages of non-viable cells were found (Figure S2). Next, we performed two sets of experiments to understand the exact mechanism by which *B. abortus* RNA down-regulates the IFN-γ-induced expression of MHC-II molecules, i.e., whether *B. abortus* RNA prevents the induction of MHC-II molecules by IFN-γ or it is able to modulate the MHC-II molecules once they are already induced by IFN-γ. We evaluate whether *B. abortus* RNA delayed the kinetic of MHC-II induction by IFN-γ and we evaluate if *B. abortus* RNA is able to down-regulate the MHC-II expression already induced by IFN-γ. For this, THP-1 cells were incubated with *B. abortus* RNA and IFN-γ for 48, 72, and 96 h. In addition, THP-1 cells were pre-incubated with IFN-γ for 24 h to induce MHC-II expression and then were stimulated with *B. abortus* RNA for additional 24 h. *B. abortus* RNA neither delays the kinetic of IFN-γ induction of MHC-II molecules (Figure S3A) nor it modifies the expression of MHC-II molecules already induced by IFN-γ (Figure S3B). Moreover, to evaluate the effects of RNA on the basal expression of MHC-II, THP-1 cells were stimulated with *B. abortus* RNA in the absence of IFN-γ. *B. abortus* RNA was not able to down-modulate the basal expression of MHC-II (Figure S3C). Altogether, these results demonstrate that *B. abortus* RNA prevents the correct induction of MHC-II molecules by IFN-γ. Then, we searched if the MHC-II down-modulation could be reproduced in primary cultures of monocytes/macrophages. To achieve this, peripheral blood-isolated human monocytes or murine bone marrow-derived macrophages (BMDM) were stimulated with *B. abortus* RNA at several doses. Afterwards, MHC-II surface expression was evaluated by flow cytometry. *B. abortus* RNA was able to significantly down-modulate the IFN-γ-induced MHC-II expression on both primary cell cultures (Figures 1E,F). Thus, *B. abortus* RNA-mediated MHC-II down-modulation could be reproduced in different monocytes/macrophages cell cultures. Finally, we evaluated whether the down-modulation of IFN-γ-induced MHC-II and MHC-I surface expression mediated by *B. abortus* RNA was an exclusive phenomenon on MHC molecules or could be extended to other IFN-γ-induced molecules. To do this, THP-1 cells were treated with *B. abortus* RNA as we previously described. Then, the expression of the co-stimulatory molecules CD40, CD86, and CD80 was evaluated by flow cytometry. The expression of CD40 and CD86 was significantly increased in *B. abortus* RNA-treated monocytes (Figures 2A,B), while it did not affect the expression of CD80 compared to the cells treated only with IFN-γ (Figure 2C). The expression of MHC-II was evaluated in parallel as a control (Figure 2D). These results demonstrate that *B. abortus* RNA-mediated MHC inhibition is specific for these molecules, since this PAMP does not alter all IFN-γ-induced molecules globally. In conclusion, these results indicate that *B. abortus* RNA is a PAMP related to bacterial viability, which is implicated in the down-modulation of IFN-γ-induced MHC-II surface expression on monocytes/macrophages observed during *B. abortus* infection.

### MHC-II Down-Modulation Could Be Extended to RNAs From Other Microorganisms

**Figure 3:**
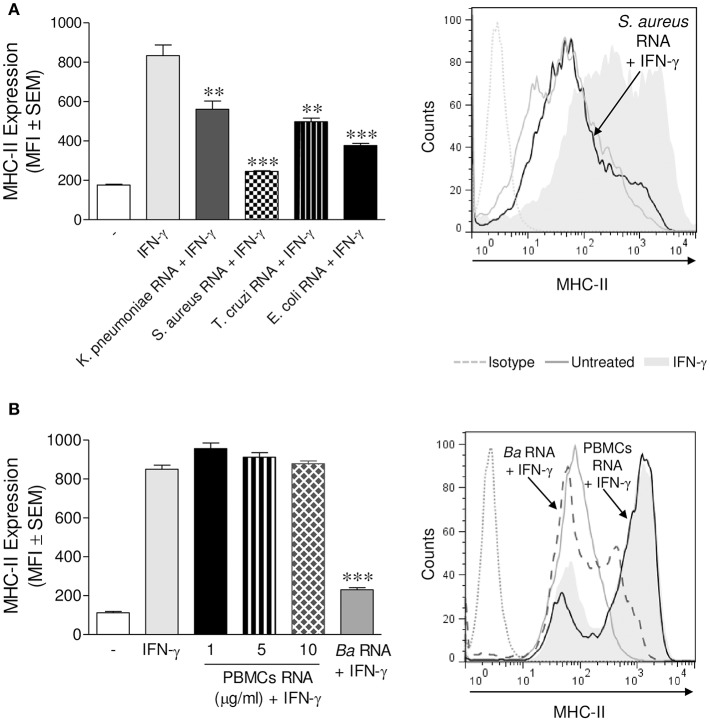
MHC-II down-modulation could be extended to RNAs from different microorganisms. **(A)** THP-1 cells were treated with RNAs from *K. pneumoniae, S. aureus, E. coli*, and *T. cruzi* (10 μg/ml) in the presence of IFN-γ for 48 h. **(B)** THP-1 cells were treated with different doses of PBMCs RNA in the presence of IFN-γ for 48 h. *B. abortus* RNA (10 μg/ml)-treated cells were used as a control. MHC-II expression was assessed by flow cytometry. Bars represent the arithmetic means ± SEM of five independent experiments. MFI, mean fluorescence intensity. ^**^P < 0.01*;* ^***^P < 0.001 vs. IFN-γ-treated cells.

We next investigated whether the ability of *B. abortus* RNA to diminish the expression of MHC-II was an exclusive characteristic of the RNA of this bacterium or if it could be extended to RNAs of other microorganisms. To elucidate this, we purified RNA from *Klebsiella pneumoniae, Staphylococcus aureus*, and *Escherichia coli*. THP-1 cells were stimulated with these RNAs plus IFN-γ for 48 h. Afterwards, MHC-II surface expression was assessed by flow cytometry. All prokaryotic RNAs evaluated were able to inhibit MHC-II surface expression (Figure 3A). Furthermore, RNA purified from a parasite (*Trypanosoma cruzi*) was also able to decrease the surface expression of MHC-II (Figure 3A). However, even the maximum dose of peripheral blood mononuclear cells (PBMCs) RNA was incapable of down-modulating MHC-II surface expression (Figure 3B). Taken together, these results show that MHC-II surface expression inhibition is not restricted to *B. abortus* RNA. Nevertheless, human RNA does not affect MHC-II expression.

### Completely Digested RNA Was Also Able to Down-Modulate MHC-II Expression

**Figure 4:**
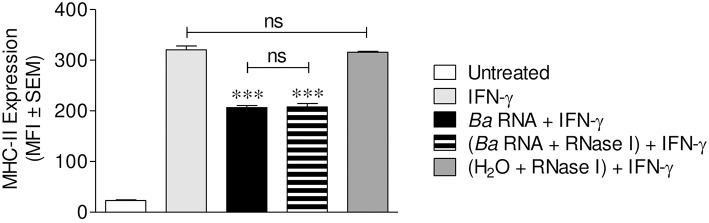
Digested-*B. abortus* RNA is also able to down-modulate MHC-II. THP-1 cells were treated with *B. abortus* RNA (10 μg/ml) or RNase I-treated *B. abortus* RNA in the presence of IFN-γ for 48 h. Cells treated with RNase I were used as a control. MHC-II expression was assessed by flow cytometry. Bars represent the arithmetic means ± SEM of five independent experiments. MFI, mean fluorescence intensity; ns, non-significant. ^***^P < 0.001 vs. IFN-γ-treated cells.

We next investigated if *B. abortus* RNA degradation products could also be implicated in MHC-II down-regulation, as they participate in MHC-I down modulation as well (14). For this, *B. abortus* RNA was treated with *E.coli* RNase I prior to stimulation of THP-1 cells in the presence of IFN-γ as we formerly described. As previously showed, the integrity of RNase-digested RNA is completely lost (14). Moreover, this digested RNA was able to down-modulate the IFN-γ-induced MHC-II surface expression to the same degree as non-digested RNA (Figure 4). As shown before, down-regulation of MHC-II was not the effect of the loss of live cells in RNase I-digested RNA stimulated cells, since this treatment did not change the percentage of viable cells (Figure S2). In addition, THP-1 cells treated with RNase I had not changes in MHC-II expression compared to IFN-γ-only-treated cells (Figure 4). Globally, these results show that *B. abortus* RNA as well as its degradation products contribute to the MHC-II down-modulation mediated by *B. abortus*.

### *B. abortus* RNA Along With Its Lipoproteins Mediate the MHC-II Down-Modulation Observed in *B. abortus* Infection

**Figure 5:**
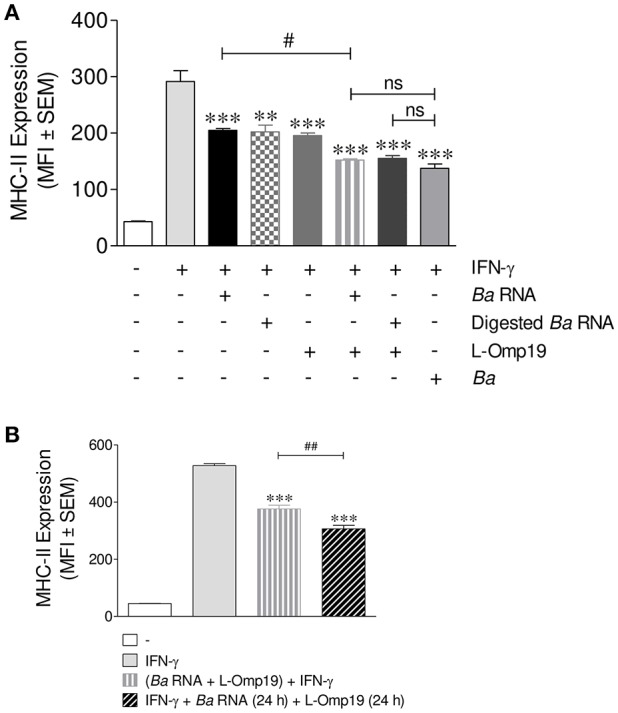
*B. abortus* RNA and *B. abortus* L-Omp19 act synergistically in MHC-II inhibition. **(A)** THP-1 cells were infected with *B. abortus* (MOI 100:1) or treated with *B. abortus* RNA (10 μg/ml), digested *B. abortus* RNA, L-Omp19 (1 μg/ml) or their combination in the presence of IFN-γ for 48 h. **(B)** THP-1 cells were treated with IFN-γ and *B. abortus* RNA (5 μg/ml) for 24 h. Afterwards, L-Omp19 (1 μg/ml) was added for other 24 h. THP-1 cells treated with both stimuli simultaneously were used as control. MHC-II expression was assessed by flow cytometry. Bars represent the arithmetic means ± SEM of five independent experiments. MFI, mean fluorescence intensity; ns, non-significant. ^**^P < 0.05; ^***^P < 0.001 vs. IFN-γ-treated cells; ^#^*P* < 0.05 vs. *Ba* RNA + IFN-γ; ^##^*P* < 0.01 vs. (*Ba* RNA + L-Omp19) + IFN-γ.

Taking into account that *B. abortus* lipoproteins (10, 13) and its RNA are components involved in the decrease of MHC-II surface expression, we wondered whether these components had a synergistic effect on MHC-II expression. For this, THP-1 cells were stimulated with *B. abortus* RNA, digested *B. abortus* RNA, the prototypical *B. abortus* lipoprotein L-Omp19 or the combination of each component in presence of IFN-γ for 48 h. MHC-II expression was assessed by flow cytometry. Again, MHC-II down-modulation was not due to the loss of viability in cells stimulated with L-Omp19 or with the combination of each component, since the percentage of viable cells did not change when compared to untreated cells (Figure S2). As it has been shown in Figure 4, *B. abortus* RNA and digested RNA were able to down-modulate MHC-II surface expression at similar levels (Figure 5A). L-Omp19 could also decrease the MHC-II expression induced by IFN-γ to the values of *B. abortus* RNA or its degradation products (Figure 5A). Furthermore, the combination of RNA and L-Omp19 induced a higher MHC-II down-modulation than merely RNA or L-Omp19 (Figure 5A). A similar effect was observed with the combination of digested *B. abortus* RNA and L-Omp19 (Figure 5A). Moreover, the combination of *B. abortus* RNA and L-Omp19 as well as digested *B. abortus* RNA and L-Omp19 showed MHC-II expression values similar to those obtained with THP-1 cells infected with *B. abortus* (Figure 5A). Taking into account that TLR8 is able to sense not only intact RNA but also RNA degradation products (23); and that we have previously demonstrated, that the down-modulation of MHC-I surface expression by intact and digested RNA is mediated by this receptor, we wanted to evaluate the participation of TLR8 in *B. abortus* RNA and L-Omp19-mediated MHC-II down-modulation. Other researchers have demonstrated that in THP-1 cells, the pre-exposure to TLR8 ligands augments the response to a posterior stimulation with TLR2 ligands (24, 25). So, to evaluate the TLR8 involvement in *B. abortus* RNA and L-Omp19-mediated MHC-II down-modulation, THP-1 cells were pre-exposed to *B. abortus* RNA and then stimulated with L-Omp19 in the presence of IFN-γ. These results were compared to those obtained with simultaneous incubation with *B. abortus* RNA and L-Omp19 in the presence of IFN-γ. We observed a greater MHC-II down-regulation in cells pre-exposed to *B. abortus* RNA and then stimulated with L-Omp19 compared to cells that received both ligands simultaneously (Figure 5B). These results demonstrated that monocytes pre-exposed to TLR8 ligands are more responsive to TLR2 ligands. Overall, these results indicate that *B. abortus* RNA (viability-associated component) and *B. abortus* lipoproteins (structural components) constitute both *Brucella* virulence factors which contribute to MHC-II down-modulation in the context of the infection.

### IFN-γ-Induced MHC-II Surface Down-Regulation Mediated by *B. abortus* RNA and Its Lipoproteins Occurs at Late Time Points During Infection

**Figure 6:**
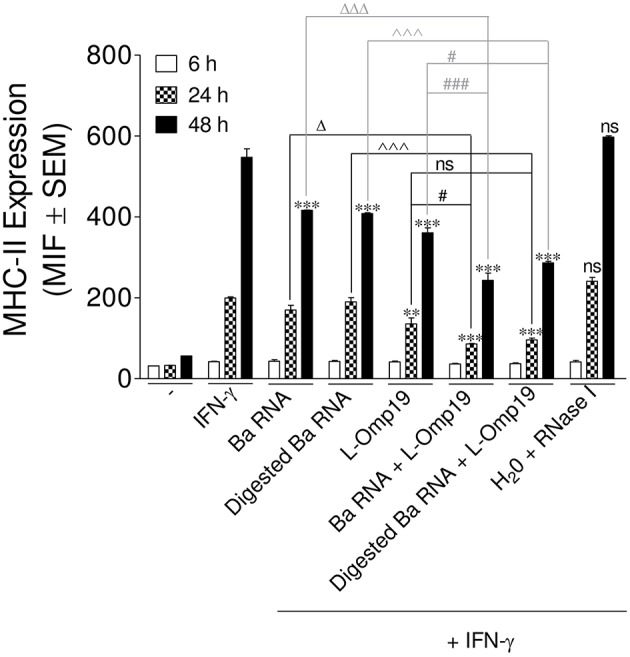
MHC-II down-modulation begins after 24 h post-stimuli. THP-1 cells were treated with *B. abortus* RNA (10 μg/ml), digested *B. abortus* RNA, L-Omp19 (1 μg/ml) or their combination in the presence of IFN-γ for 6, 24, and 48 h. Cells treated with RNase I were used as a control. MHC-II expression was assessed by flow cytometry. Bars represent the arithmetic means ± SEM of five independent experiments. MFI, mean fluorescence intensity; ns, non-significant. ^**^P < 0.01; ^***^P < 0.001 vs. IFN-γ-treated cells; ^Δ^*P* < 0.05; ^ΔΔΔ^*P* < 0.001 vs. *Ba* RNA; ^∧∧∧^*P* < 0.001 vs. Digested *Ba* RNA; ^#^*P* < 0.05; ^###^*P* < 0.001 vs. L-Omp19.

Next, we wanted to evaluate the mechanism by which *B. abortus* RNA alone or together with lipoproteins was down-modulating MHC-II expression. Therefore, at first, we evaluated MHC-II down-regulation kinetics. In order to perform this, THP-1 cells were stimulated as previously described, with *B. abortus* RNA, RNase I-treated *B. abortus* RNA, the *B. abortus* lipoprotein L-Omp19 or the combination of each component in the presence of IFN-γ. At 6, 24, and 48 h, the surface expression of MHC-II was evaluated by flow cytometry. At 6 h, there is neither up-regulation of MHC-II on IFN-γ-treated cells nor MHC-II modulation with the different stimuli. However, at 24 h MHC-II expression on IFN-γ-treated cells is induced, being even accentuated at 48 h. At 24 h, L-Omp19 (but not *B. abortus* RNA and digested *B. abortus* RNA) significantly down-regulated MHC-II surface expression. Meanwhile at 48 h, *B. abortus* RNA, digested *B. abortus* RNA and L-Omp19 were able to significantly inhibit the MHC-II surface expression (Figure 6). Furthermore, at both times, as shown in Figure 5A, the combination of RNA (or digested *B. abortus* RNA) plus L-Omp19 induced a higher MHC-II down-modulation than merely RNA, digested RNA or L-Omp19 (Figure 6). We also demonstrate that *B. abortus* RNA induced the expression of MHC-II on DCs (Figure S4A) and down-regulated the LPS-induced MHC-II expression on human monocytes (Figure S4B). Overall, these results demonstrate that *B. abortus* RNA, digested *B. abortus* RNA, and L-Omp19 alone or together decrease MHC-II only when these molecules are induced by a MHC-II up-regulator, such as IFN-γ. In turn, they show that when the induction of MHC-II mediated by IFN-γ is the highest (which is observed after 48 h post stimulation) the greater the degree of MHC-II surface inhibition generated by the components.

### *B. abortus* RNA Along With Its Lipoproteins Decrease MHC-II Surface Expression Predominantly by a Mechanism of Inhibition of MHC-II Expression

**Figure 7:**
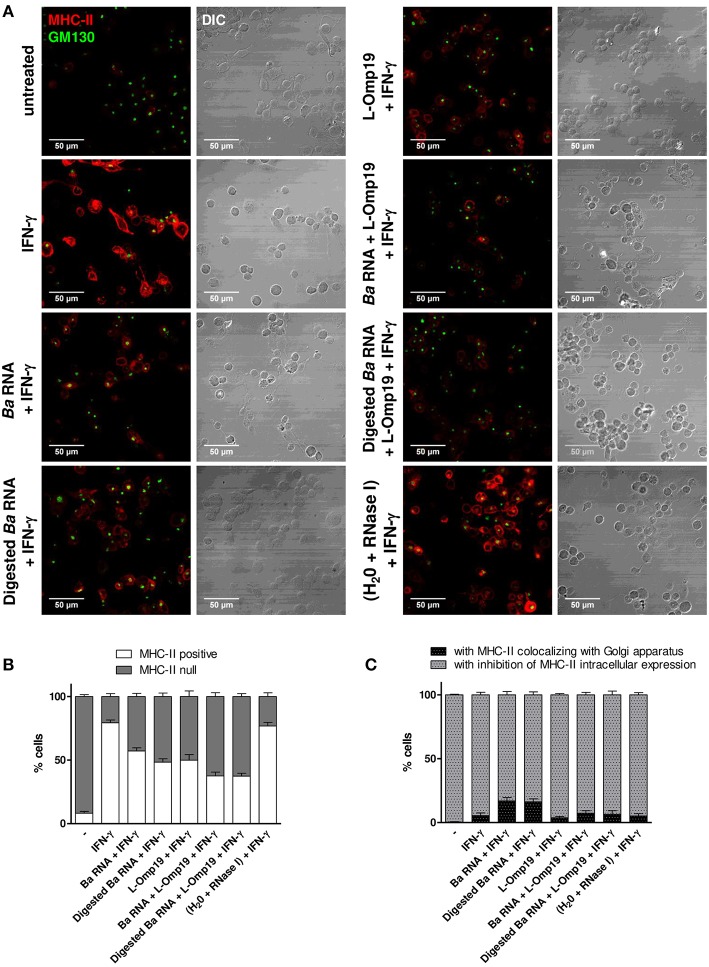
*B. abortus* RNA and digested *B. abortus* RNA inhibit MHC-II expression on and inside the cells. **(A)** Confocal micrographs of THP-1 cells treated with *B. abortus* RNA (10 μg/ml), digested *B. abortus* RNA, L-Omp19 (1 μg/ml) or their combination in the presence of IFN-γ for 48 h. Cells treated with RNase I were used as a control. MHC-II was detected with a primary anti-human MHC-II Ab (L243) followed by Alexa 546-labeled secondary Ab (red). Golgi apparatus was detected using a mAb specific for GM130 followed by Alexa 488-labeled secondary Ab (green). Results are representative of three independent experiments. **(B)** Quantification of cells expressing MHC-II (MHC-II positive cells) or not (MHC-II null). Data is expressed as the percentage of cells ± SEM of three independent experiments. **(C)** Quantification of null cells with MHC-II colocalizing with Golgi apparatus or with inhibition of intracellular MHC-II expression. Data is expressed as the percentage of cells ± SEM of three independent experiments. The number of cells counted per experimental group was 200. DIC, differential interference contrast.

Once the kinetics of MHC-II down modulation has been established, we then wondered the mechanism of this phenomenon. We have previously shown that lipoproteins of *B. abortus* decreased MHC-II expression by inhibiting MHC-II genes transcription (13). More recently, we demonstrated that *B. abortus* RNA down-modulates MHC-I by retaining them inside the Golgi apparatus rather than by transcriptional inhibition as occurred with MHC-II (14). So, we wanted to understand why *B. abortus* RNA alone or together with lipoproteins is able to down-modulate MHC-II. To do this, THP-1 cells were stimulated with *B. abortus* RNA, RNase I-treated *B. abortus* RNA, L-Omp19 or the combination of each component in the presence of IFN-γ for 6, 24, and 48 h. Then, MHC-II expression and localization were evaluated by confocal microscopy. The expression of MHC-II was determined with an anti-human MHC-II mAb followed by Alexa 546-labeled secondary antibody. Golgi apparatus was detected using a monoclonal antibody specific for GM130 followed by Alexa 488-labeled secondary Ab. In accordance with the kinetics results, at 6 h almost no MHC-II expression is observed under any condition (data not shown). At 24 h, IFN-γ-treated cells showed up-regulation of MHC-II surface expression. However, there was not a significant reduction on MHC-II expression mediated by *B. abortus* RNA, digested *B. abortus* RNA, L-Omp19 or their combination (data not shown). At 48 h, two populations were observed: cells with MHC-II expression confined to the cellular surface (named MHC-II-positive cells) and cells with no MHC-II expression on the cellular surface (named MHC-II null cells). As expected, the majority of the cells were MHC-II positive in IFN-γ-treated cells (Figures 7A,B and Figure S5). When cells were treated with *B. abortus* RNA and digested RNA, there was an increase in the number of MHC-II null cells. The same phenomenon was observed with L-Omp19. In addition, when the components were combined, the percentage of MHC-II null cells was even higher. As expected, the cells treated with IFN-γ and merely RNase I behaved similarly to those treated only with IFN-γ (Figures 7A,B).

Given the fact that MHC-II null cells are responsible for the down-modulation in MHC-II surface expression that we previously observed in the flow cytometry experiments, we wondered what mechanisms were causing this absence of MHC-II surface expression. Consequently, inside the MHC-II null population, we calculated the percentage of cells with MHC-II retained within the Golgi apparatus and the percentage of cells with no expression of MHC-II inside the cell at all. Cells treated with *B. abortus* RNA and digested RNA showed mainly a reduction in MHC-II intracellular expression although 17% of cells had MHC-II retained in Golgi apparatus (Figures 7A,C and Figure S5). However, when *B. abortus* RNA or digested RNA were combined with L-Omp19, the percentage of Golgi apparatus retention decreases to the values obtained for L-Omp19 alone (Figures 7A,C).

Taken together, these results demonstrate that there are two possible mechanisms involved in the down-modulation of MHC-II surface expression on monocytes/macrophages. However, the inhibition of MHC-II expression is the prevalent mechanism when cells are treated with *B. abortus* RNA together with lipoproteins.

### IL-6 Is Involved in MHC-II Down-Modulation Mediated by *B. abortus* RNA and Its Lipoproteins

**Figure 8:**
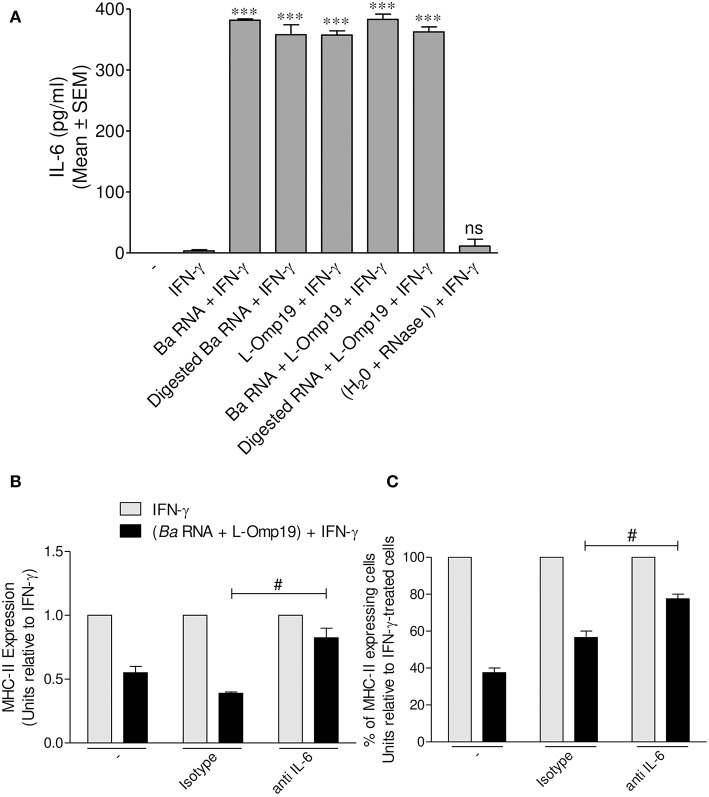
IL-6 is a soluble mediator involved in *B. abortus* RNA and L-Omp19-mediated MHC-II down-modulation. **(A)** THP-1 cells were treated with *B. abortus* RNA (10 μg/ml), digested *B. abortus* RNA, L-Omp19 (1 μg/ml) or their combination in the presence of IFN-γ for 48 h. Then, supernatants were harvested and IL-6 secretion was quantified by ELISA sandwich. **(B,C)** THP-1 cells were treated with *B. abortus* RNA (10 μg/ml) and L-Omp19 (1 μg/ml) in the presence of IFN-γ and in the presence of neutralizing anti-IL-6 or its isotype control for 48 h. MHC-II expression was assessed by flow cytometry. **(B)** Bars represent the arithmetic means ± SEM of MHC-II positive cells corresponding to three independent experiments. **(C)** Quantification of cells expressing MHC-II (MHC-II positive cells). Data is expressed as the percentage of cells relative to IFN-γ ± SEM of three independent experiments. MFI, mean fluorescence intensity; ns, non-significant. ^***^P < 0.001 vs. IFN-γ-treated cells; ^#^*P* < 0.05 vs. isotype control.

*B. abortus* lipoproteins mediate the down-regulation of MHC-II genes transcription, at least in part, via IL-6, as we previously reported (13). As consequence, and given the fact that the main mechanism by which *B. abortus* RNA (alone or together with lipoproteins) down-modulates MHC-II surface expression is the inhibition of MHC-II inside the cell, we tested whether IL-6 could be a possible mediator in this phenomenon. In order to perform this, THP-1 monocytes were stimulated with *B. abortus* RNA, RNase I-treated *B. abortus* RNA, L-Omp19 or a combination of each component in the presence of IFN-γ for 48 h. Afterwards, secreted IL-6 was measured in the supernatants by ELISA sandwich. As previously demonstrated, L-Omp19 stimulates the secretion of IL-6 (Figure 8A). This cytokine was induced by *B. abortus* RNA and digested *B. abortus* RNA as well (Figure 8A). Additionally, we stimulated THP-1 monocytes with the combination of *B. abortus* RNA, L-Omp19, and IFN-γ for 48 h in presence of an IL-6 neutralizing antibody or the isotype control. There was a partial recovery of the inhibition of IFN-γ-induced MHC-II expression when IL-6 was neutralized (Figure 8B). We also observed that the treatment with *B. abortus* RNA plus IFN-γ in the presence of anti-IL-6 not only increases the MFI of MHC-II molecules within the MHC-II-expressing cell population but also slightly increases the percentage of cells expressing MHC-II (Figure 8C). Therefore, this cytokine is one of the soluble mediators implicated in MHC-II down-modulation by *B. abortus* RNA and lipoproteins.

### MHC-II Surface Inhibition Mediated by *B*. *abortus* RNA Alone or Together With Its Lipoproteins Correlates With Reduced Antigen Presentation to CD4^+^ T Cells

**Figure 9:**
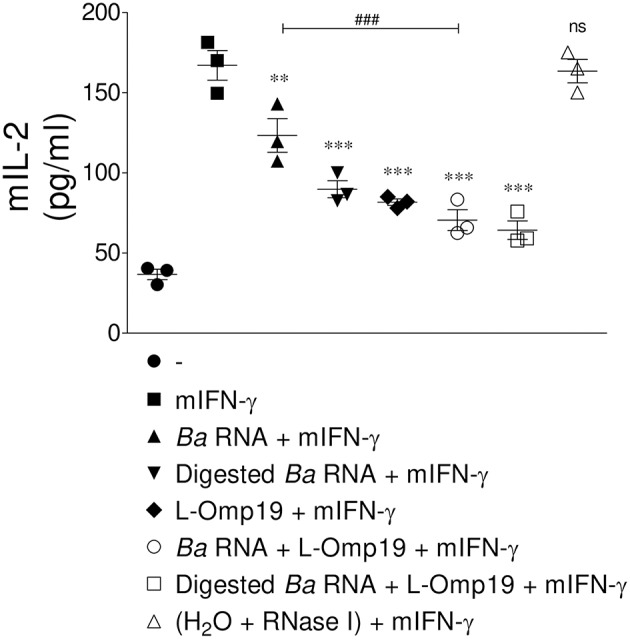
MHC-II down-modulation correlates with diminished antigen presentation to CD4^+^ T cells. BMM were treated with *B. abortus* RNA (10 μg/ml), digested *B. abortus* RNA, L-Omp19 (1 μg/ml) or their combination in the presence of mIFN-γ for 48 h. Then, cells were washed and incubated with 100 μg/ml of OVA peptide for 3 h at 37°C. Afterwards, cells were washed and co-cultured for 20 h at 37°C with BO97.10 cells, a T cell hybridoma specific for OVA peptide. T cell activation was measured by quantifying mIL-2 secretion in culture supernatants. ^**^P < 0.01; ^***^P < 0.001 vs. mIFN-γ-treated cells; ns, non-significant; ^###^*P* < 0.001 vs. *Ba* RNA.

At last, we evaluate whether the down-regulation of MHC-II surface expression triggered by *B. abortus* RNA alone or together with lipoproteins had a functional correlation. For this purpose, we performed an antigen presentation assayusing a I-Ab-restricted T cell hybridoma specific for OVA 323-339 peptide (BO97.10). Murine BMDM were stimulated with *B. abortus* RNA, digested *B. abortus* RNA, L-Omp19 or the combination of each component in the presence of mIFN-γ (murine IFN-γ). After 48 h, BMDM were incubated with OVA peptide and the T cell hybridoma BO97.10. As shown in Figure 9, the treatment with mIFN-γ induced the presentation of OVA peptide at 20 h, as supported by the capacity of BMDM to induce the secretion of IL-2 by BO97.10 cells. However, BMDM treated with *B. abortus* RNA, digested *B. abortus* RNA, L-Omp19 or the combination of each component plus mIFN-γ showed a significantly reduced capacity of OVA peptide presentation, as evidenced by decreased response of BO97.10 cells, related to BMDM treated only with mIFN-γ (Figure 9). As expected, BMDM treated with mIFN-γ and merely RNase I behaved similarly to those treated only with mIFN-γ (Figure 9). Overall, these results demonstrated that MHC-II down-regulation mediated by *B. abortus* RNA alone or together with lipoproteins is biologically relevant as it directly correlates with reduced antigen presentation to MHC-II-restricted CD4^+^ T cells.

## Discussion

CD4^+^ T cells are central to host resistance to *Brucella* infection (6–9). The secretion of IFN-γ by these cells enhances the bactericidal activity of *Brucella*-infected macrophages. Also, IFN-γ has an important role in activating macrophages. It enhances MHC-II expression on their surfaces, which results in increased antigen presentation to CD4^+^ T cells (26–28). MHC-II genetic deficiency altering CD4^+^ T lymphocytes completely impairs *Brucella* control in lungs, liver and spleen (29), highlighting the crucial role of these cells in the development of a protective response against infection. However, when bacteria are not fully eliminated, they are able to hide inside macrophages evading their eradication by the host immune system. One mechanism that would explain how *Brucella* is able to persist in the host chronically is the inhibition of MHC-II-restricted antigen presentation on *Brucella*-infected macrophages. We previously demonstrated that *B. abortus* infection down-regulates IFN-γ-induced MHC-II surface expression on monocytes/macrophages (10). Moreover, this down-modulation is caused by *B. abortus* lipoproteins (10, 13). However, these bacterial structural components were less efficient at reducing MHC-II surface expression than live bacteria, suggesting that another component related to bacterial viability must be involved in this phenomenon. In this study we demonstrated that *B. abortus* RNA, a component associated with viable bacteria, participates in the down-regulation of IFN-γ-induced expression of MHC-II molecules on monocytes/macrophages. The MHC-II surface inhibition was not the result of a detrimental effect of the RNA on monocytes (i.e., apoptosis or necrosis). Our results also demonstrate that *B. abortus* RNA neither delays the kinetic of IFN-γ induction of MHC-II molecules nor it modifies the expression of MHC-II molecules already induced by IFN-γ. On the contrary, *B. abortus* RNA prevents the correct induction of MHC-II molecules by IFN-γ. Moreover, MHC-II down-regulation was observed on cells of the monocytic line THP-1, on peripheral blood-purified human monocytes, and murine bone marrow-derived macrophages.

We have previously demonstrated that *B. abortus* RNA is also a component involved in *B. abortus*-mediated MHC-I surface inhibition (14). However, the inhibition of MHC molecules surface expression is not due to a global effect on IFN-γ-induced molecules. On the contrary, *B. abortus* RNA up-regulated the IFN-γ-induced expression of the co-stimulatory molecules CD40 and CD86, while it did not modify the expression of CD80. We also observed that MHC-II surface inhibition is not exclusive for *B. abortus* as it could be extrapolated to RNAs of other bacteria. Moreover, the phenomenon was observed with the RNA of the parasite *T. cruzi*. These results suggest that this singular immune regulation could happen in the context of other infectious processes.

Some years ago, it was demonstrated that the immune system is able to sense RNA degradation products through TLR8 (23). The fact that TLR8 recognizes degradation products nurtures the concept that bacterial or human phosphatases or nucleases might act before the activation of this receptor, as it has been previously proposed (30), phenomenon which could be compared to the prerequisite of the action of DNAse II in order to activate TLR9 (31, 32). In agreement with this idea, our results showed that *E.coli* RNase I-treated *B. abortus* RNA diminished MHC-II expression to the same extent as non-digested RNA. Therefore, our evidences suggest that the down-modulation of MHC-II by RNA and its degradation products is mediated by TLR8. In line with this, we recently published that the down-modulation of MHC-I surface expression by intact and digested RNA is mediated by hTLR8/mTLR7 as was demonstrated using BMDM from TLR7 [the TLR that acts as TLR8 in mice (33–35)] KO mice (14).

As we have previously demonstrated, HKBA was also capable of inhibiting MHC-II surface expression (10). Furthermore, *B. abortus* lipoproteins (structural components present in HKBA) were able to mimic the MHC-II down-modulation mediated by HKBA (10, 13). However, HKBA or *B. abortus* lipoproteins generated lower MHC-II reduction than live bacteria. Based on the evidences found in this study, we can now understand the differences in MHC-II down-modulation. Taking into account that RNA is rapidly eliminated when the bacteria lose their viability (14, 16, 17), in our previous experiments with HKBA, the MHC-II surface down-modulation was only mediated by lipoproteins. On the other hand, in the context of the infection, MHC-II surface down-modulation was not only mediated by lipoproteins but also by RNA. In line with this, we demonstrated that the combination of RNA and L-Omp19 induced higher MHC-II down-modulation than RNA or L-Omp19 alone. The synergism between RNA and lipoproteins can be understood in terms of cross-talk between TLRs. Immune responses to viral and bacterial pathogens depend on activation of intricate TLR-TLR interactions (36, 37). Stimulation of TLR8 alongside with TLR3 or TLR4 ligands on macrophages or DCs provoke a synergistic effect on activation of NF-kB and IFN regulatory factor (IRF), as it has been recently shown (36, 38). Bearing in mind this idea, Cervantes et al. showed that in human monocytes after TLR8 activation, the expression of TLR2 is induced (24). Other researchers have demonstrated that in THP-1 cells, the pre-exposure to 3M-002 (TLR8 ligand) augments the response to a posterior stimulation with TLR2 ligands (25). Another interesting finding is that CD14 is up regulated in monocytes that are differentiating to DCs when they are exposed to R848 (TLR7/8 ligand) (39). It has been described that CD14 facilitates the signaling through TLR2 mediated by bacterial lipoproteins (40–42). Our experiments of pre-exposure of THP-1 cells with *B. abortus* RNA before L-Omp19 (TLR2 ligand) corroborate in a functional way the involvement of TLR8 in MHC-II down-modulation.

Our kinetic studies demonstrated that MHC-II down-modulation by *B. abortus* RNA alone or in combination with *B. abortus* lipoproteins begins after 24 h post-stimuli, when MHC-II expression was induced by effect of IFN-γ. However, the maximum MHC-II surface down-modulation was observed at 48 h, in the moment of highest induction by IFN-γ. In turn, in agreement with previously published results (13, 43), *B. abortus* RNA alone or in combination with *B. abortus* lipoproteins is incapable of modulating MHC-II basal expression on monocytes. Our results also demonstrate that *B. abortus* RNA induced the expression of MHC-II on DCs. These results agree with our published results showing that *B. abortus* infection induces DCs maturation, as evidenced by the up-regulation of CD86, CD80, CCR7, CD83, MHC-II, MHC-I, and CD40 at 24 h post-infection (20). The apparent discrepancies between the MHC-II up-regulation in DCs and the down-regulation in monocytes/macrophages could be explain in terms of the kinetics of *Brucella* infection. One explanation is that activation of DCs with *B. abortus* is likely relevant at the onset of immune response, when Th1 and T CD8^+^ responses are triggered. At later time points *Brucella* might be able to circumvent these responses to establish a chronic infection by means of different evasion mechanism such as down-modulation of MHC-II molecules in macrophages, where it dwells (12). Th1 cells and a high concentration of IFN-γ, a key cytokine in the induction of MHC-II expression, antigen processing and presentation by macrophages, integrate the immune response elicited against *Brucella*. However, in light of the obtained results, *B. abortus* might potentially inhibit the expression of MHC-II molecules regardless the triggering stimulus. In turn, our results suggest that the phenomenon could occur in the absence of an established production of IFN-γ, i.e., before the activation of the Th1 response, at early stages of *B. abortus* infection (with LPS as possible inducer of MHC-II). Nevertheless, MHC-II down-modulation is more pronounced in the moment of infection in which adaptive immunity begins to be relevant, i.e., when IFN-γ secreted by T lymphocytes is stimulating the presentation of *Brucella* antigens to MHC-II-restricted CD4^+^ T cells.

One issue that merits discussion is the mechanism by which *B. abortus* RNA alone or in combination with lipoproteins is able to down-modulate MHC-II. We previously demonstrated that *B. abortus* lipoproteins down-regulate the IFN-γ-induced MHC-II surface expression by decreasing the transcription of MHC-II mRNA with the consequent inhibition of protein synthesis (13). Years later we demonstrated that *B. abortus* RNA decreases MHC-I surface expression by retaining these molecules within the Golgi apparatus (14). However, to our surprise, with *B. abortus* RNA we observed only a small percentage of cells with MHC-II molecules retained in the Golgi apparatus being the main mechanism the inhibition of the intracellular expression of these molecules. Regarding *B. abortus* lipoproteins, as expected, only intracellular inhibition of MHC-II expression was observed. On the other hand, when *B. abortus* RNA was combined with lipoproteins, the Golgi apparatus retention observed with merely RNA decreased, constituting MHC-II intracellular inhibition the principal mechanism. The confocal micrographs of *B. abortus* RNA plus lipoproteins allow us to evoke and understand what we previously observed in *B. abortus*-infected monocytes: a drastic reduction of MHC-II expression either in the surface or within the cell (11). Taken together, our results show that even for the treatment with *B. abortus* RNA alone, the predominant mechanism is the one previously described for lipoproteins. This mechanism is consistent with our kinetic experiments in which we observed a more pronounced inhibition at 48 h post-stimulation.

Another relevant question addressed in this study was the possible soluble mediators involved in the down-regulation of IFN-γ-induced MHC-II surface expression. We have demonstrated that IL-6 contributes to the inhibition of MHC-II expression mediated by *B. abortus* infection and their lipoproteins (10). Furthermore, we could discard the participation of IL-10 in this phenomenon (10). More recently, we demonstrated that *B. abortus* lipoproteins decrease the transcription of MHC-II and CIITA mRNA, and IRF-1 (regulatory transcriptional factor for CIITA) expression, trough IL-6 (13). On the other hand, we have recently demonstrated that *B. abortus* and its RNA induce the retention of MHC-I molecules within Golgi apparatus through EGFR signaling pathway (14, 15). Given that we demonstrated that the predominant mechanism of MHC-II surface down-modulation is the inhibition of expression of these molecules, we focus our attention on IL-6. Our results demonstrated that IL-6 is one soluble factor that participates in MHC-II down-regulation mediated by the combination of *B. abortus* RNA and lipoproteins. However, given the partial reversion of MHC-II surface down-modulation mediated by neutralizing Ab to IL-6, these results do not rule out that the EGFR pathway may also be involved. This pathway would explain the Golgi apparatus retention observed in a small percentage of cells.

Although we demonstrated that *B. abortus* RNA decreases MHC-II surface expression, it increases the expression of co-stimulatory molecules (CD40 and CD80). However, the global effect is the reduction of antigen presentation of *B. abortus* RNA stimulated-macrophages to CD4^+^ T cells, which is an important functional correlation to our study. Moreover, *B. abortus* RNA in combination with lipoproteins leads to a lower antigen presentation.

Finally, we have elucidated that *B. abortus* RNA is a component associated to bacterial viability that along with lipoproteins participates in MHC-II surface down-modulation. Accordingly, through this phenomenon, the bacteria could prevent the CD4^+^ T cell recognition in order to evade host immune response.

## Data Availability

The datasets generated for this study are available on request to the corresponding author.

## Ethics Statement

The studies involving human participants were reviewed and approved by Ethical Committee of the IMEX Institute. The patients/participants provided their written informed consent to participate in this study. The animal study was reviewed and approved by Animal Care and Use Committee of the IMEX Institute.

## Author Contributions

The experiments were designed and conceived by MM, GG, and PB. The experiments were performed by MM, AT, AS, JM, FM, JA, MG, and LB. Data was analyzed by MM. Materials, reagents were facilitated by SO and GG. They provided key suggestions to this work as well. All experiments were supervised by PB. The interpretation of data and the preparation of the manuscript were performed by MM and PB. The manuscript was reviewed by all authors.

### Conflict of Interest Statement

The authors declare that the research was conducted in the absence of any commercial or financial relationships that could be construed as a potential conflict of interest.

## Supplementary Material

Figure S1 Potential RNA contaminants are not involved in MHC-II down-modulation. **(A,B)** THP-1 cells were stimulated with DNase **(A)** or PK **(B)**-treated *B. abortus* RNA in the presence of IFN-γ for 48 h. Cells treated with DNase or PK alone were used as negative controls. Cells treated with *B. abortus* RNA were used as positive controls. MHC-II expression was assessed by flow cytometry. Bars represent the arithmetic means ± SEM of three independent experiments. MFI, mean fluorescence intensity; ^*^*P* < 0.05; ^**^*P* < 0.01; ^***^*P* < 0.001 vs. IFN-γ-treated cells; ^###^*P* < 0.001 vs. negative controls.

Figure S2 MHC-II down-modulation is not due to a loss of cell viability. THP-1 cells were treated with *B. abortus* RNA (10 μg/ml), L-Omp19 (1 μg/ml), RNase I-treated *B. abortus* RNA, or a combination of each component in the presence of IFN-γ for 48 h. Then the percentage of 7AAD^+^ cells were evaluated. Cells treated with Paraformaldehyde (PFA) were used as a positive control of the technique. ^###^*P* < 0.001 vs. untreated cells.

Figure S3 *B. abortus* RNA prevents the induction by IFN-γ of MHC-II. **(A)** THP-1 cells were treated with *B. abortus* RNA (5 μg/ml) in the presence of IFN-γ for 48, 72, or 96 h. **(B)** THP-1 cells were treated with IFN-γ for 24 h and then *B. abortus* RNA was added for other 24 h. **(C)** THP-1 cells were treated with *B. abortus* RNA for 48 h. MHC-II expression was assessed by flow cytometry. Bars represent the arithmetic means ± SEM of three independent experiments. MFI, mean fluorescence intensity; ns, non-significant; ^*^*P* < 0.05; ^**^*P* < 0.01; ^***^*P* < 0.001 vs. IFN-γ-treated cells; ^###^*P* < 0.001 vs. (*Ba* RNA + IFN-γ).

Figure S4 *B. abortus* RNA induced MHC-II expression on DCs while it inhibits the LPS-induced MHC-II on human monocytes. **(A)** DCs were treated with *B. abortus* RNA (1–10 μg/ml) or *E. coli* LPS (10 ng/ml) as a positive control of MHC-II induction for 24 h. **(B)** THP-1 cells were treated with *B. abortus* RNA (5 μg/ml) in the presence of *E coli* LPS (10 ng/ml) for 48 h. MHC-II expression was assessed by flow cytometry. Bars represent the arithmetic means ± SEM of three independent experiments. MFI, mean fluorescence intensity; ^#^*P* < 0.05; ^##^*P* < 0.01; ^###^*P* < 0.001 vs. untreated cells; ^*^*P* < 0.05 vs. LPS-treated cells.

Figure S5 *B. abortus* RNA and lipoproteins down-modulate MHC-II mainly by MHC-II inhibition inside the cells. Zooms of confocal micrographs of THP-1 cells treated with *B. abortus* RNA (10 μg/ml) or *B. abortus* RNA (10 μg/ml) plus L-Omp19 (1 μg/ml) in the presence of IFN-γ, as representative figures of MHC-II down-modulation mechanisms (retention in Golgi apparatus and MHC-II inhibition). MHC-II was detected with a primary anti-human MHC-II Ab (L243) followed by Alexa 546-labeled secondary Ab (red). Golgi apparatus was detected using a mAb specific for GM130 followed by Alexa 488-labeled secondary Ab (green). DIC, differential interference contrast.
