# Supplementary figures and images for: Bacterial RNA Contributes to the Down-Modulation of MHC-II Expression on Monocytes/Macrophages Diminishing CD4+ T Cell Responses
Source: Front Immunol. 2019 Sep 13;10:2181. doi: 10.3389/fimmu.2019.02181 (PMC6753364; doi:10.3389/fimmu.2019.02181)

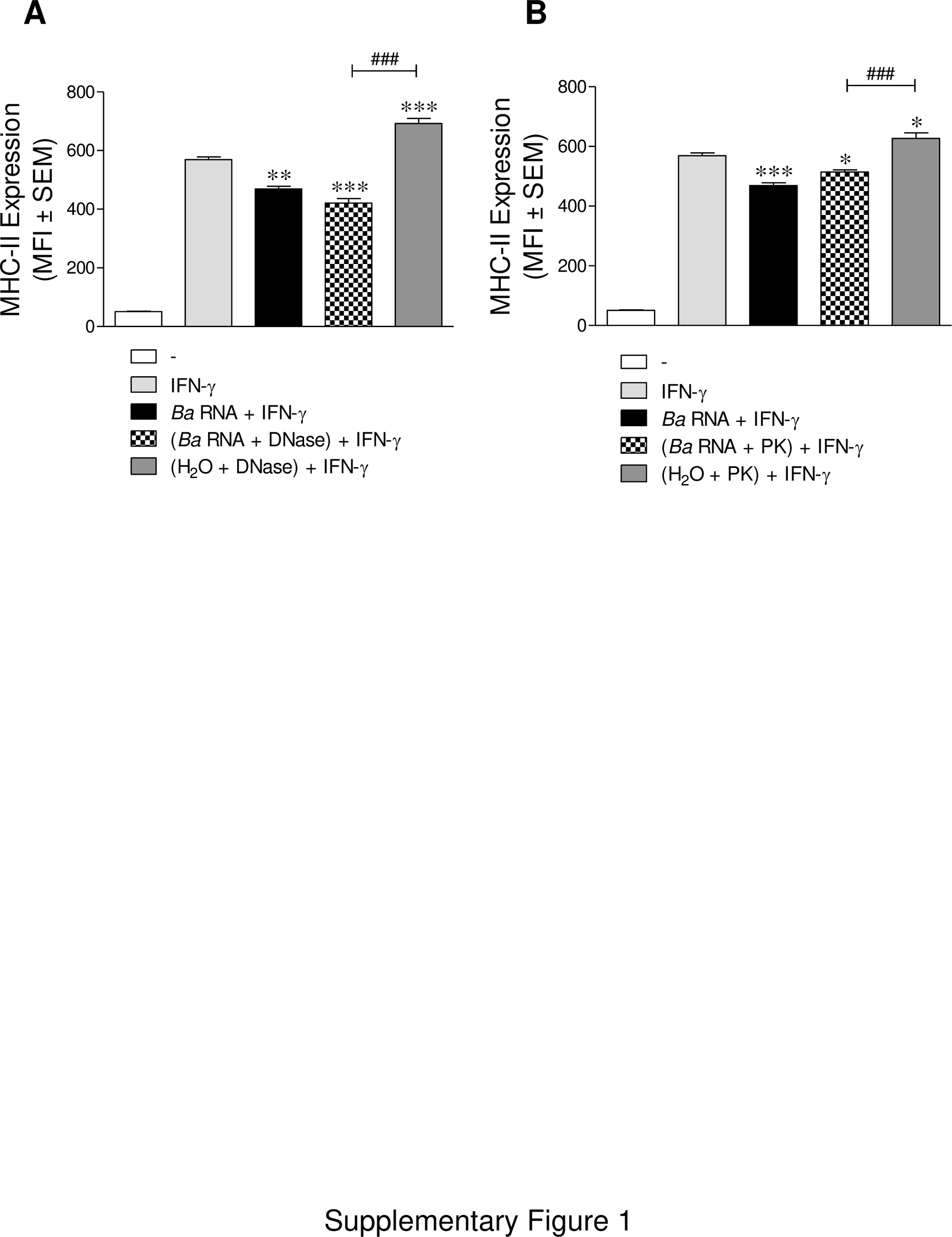

Supplement: Figure S1 — Potential RNA contaminants are not involved in MHC-II down-modulation. (A,B) THP-1 cells were stimulated with DNase (A) or PK (B)-treated B. abortus RNA in the presence of IFN-γ for 48 h. Cells treated with DNase or PK alone were used as negative controls. Cells treated with B. abortus RNA were used as positive controls. MHC-II expression was assessed by flow cytometry. Bars represent the arithmetic means ± SEM of three independent experiments. MFI, mean fluorescence intensity; *P < 0.05; **P < 0.01; ***P < 0.001 vs. IFN-γ-treated cells; ###P < 0.001 vs. negative controls. [file Image_1.TIF]

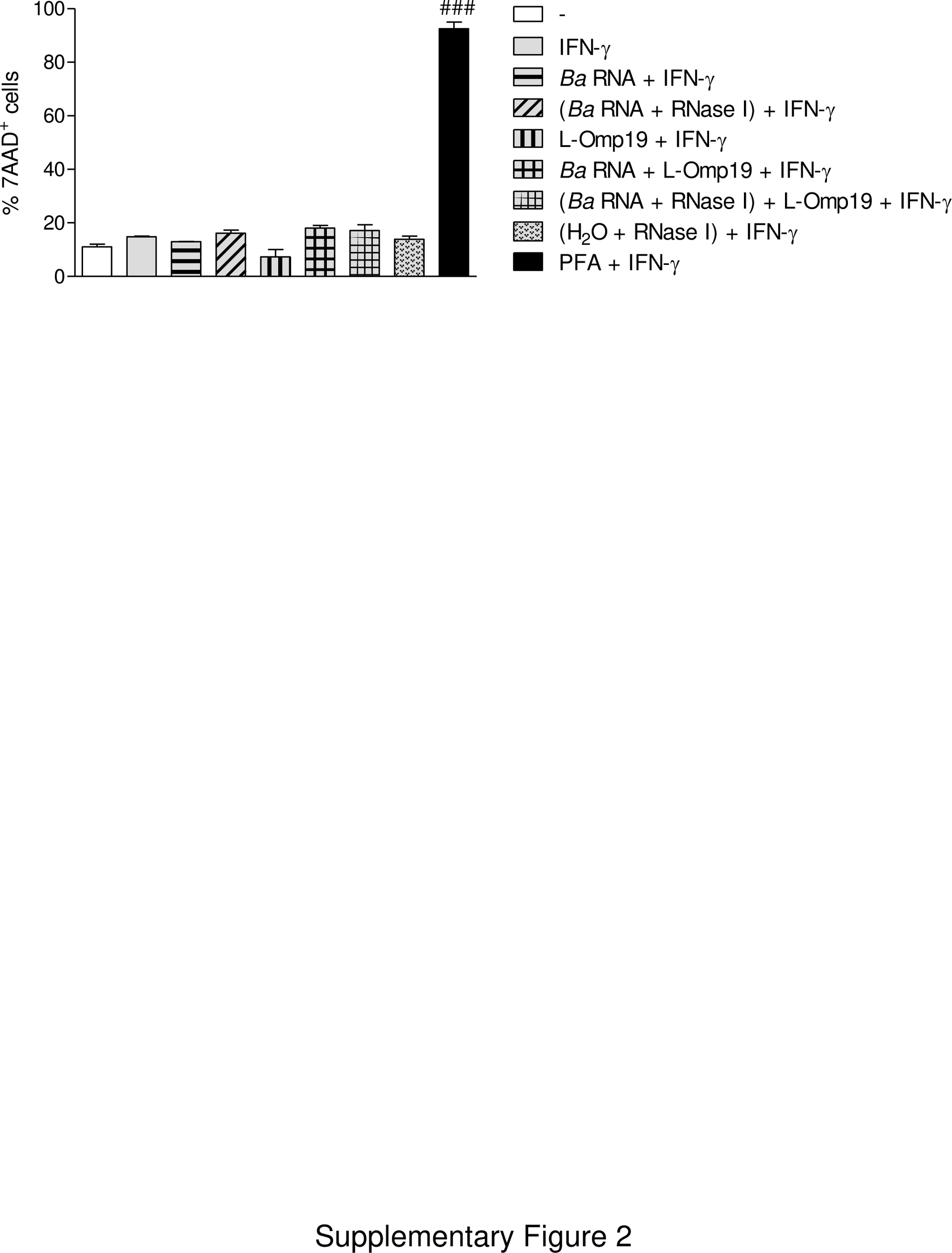

Supplement: Figure S2 — MHC-II down-modulation is not due to a loss of cell viability. THP-1 cells were treated with B. abortus RNA (10 μg/ml), L-Omp19 (1 μg/ml), RNase I-treated B. abortus RNA, or a combination of each component in the presence of IFN-γ for 48 h. Then the percentage of 7AAD+ cells were evaluated. Cells treated with Paraformaldehyde (PFA) were used as a positive control of the technique. ###P < 0.001 vs. untreated cells. [file Image_2.TIF]

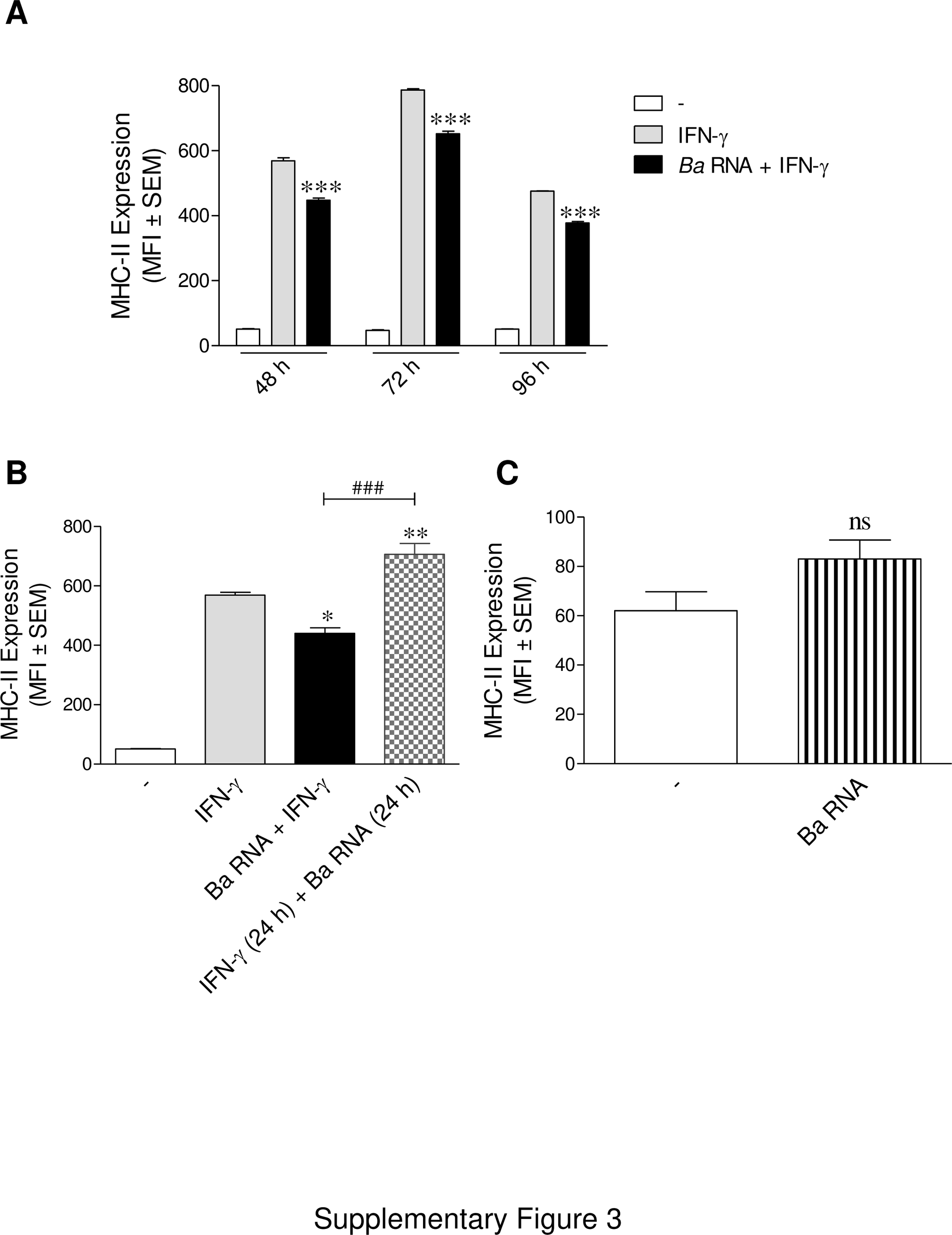

Supplement: Figure S3 — B. abortus RNA prevents the induction by IFN-γ of MHC-II. (A) THP-1 cells were treated with B. abortus RNA (5 μg/ml) in the presence of IFN-γ for 48, 72, or 96 h. (B) THP-1 cells were treated with IFN-γ for 24 h and then B. abortus RNA was added for other 24 h. (C) THP-1 cells were treated with B. abortus RNA for 48 h. MHC-II expression was assessed by flow cytometry. Bars represent the arithmetic means ± SEM of three independent experiments. MFI, mean fluorescence intensity; ns, non-significant; *P < 0.05; **P < 0.01; ***P < 0.001 vs. IFN-γ-treated cells; ###P < 0.001 vs. (Ba RNA + IFN-γ). [file Image_3.TIF]

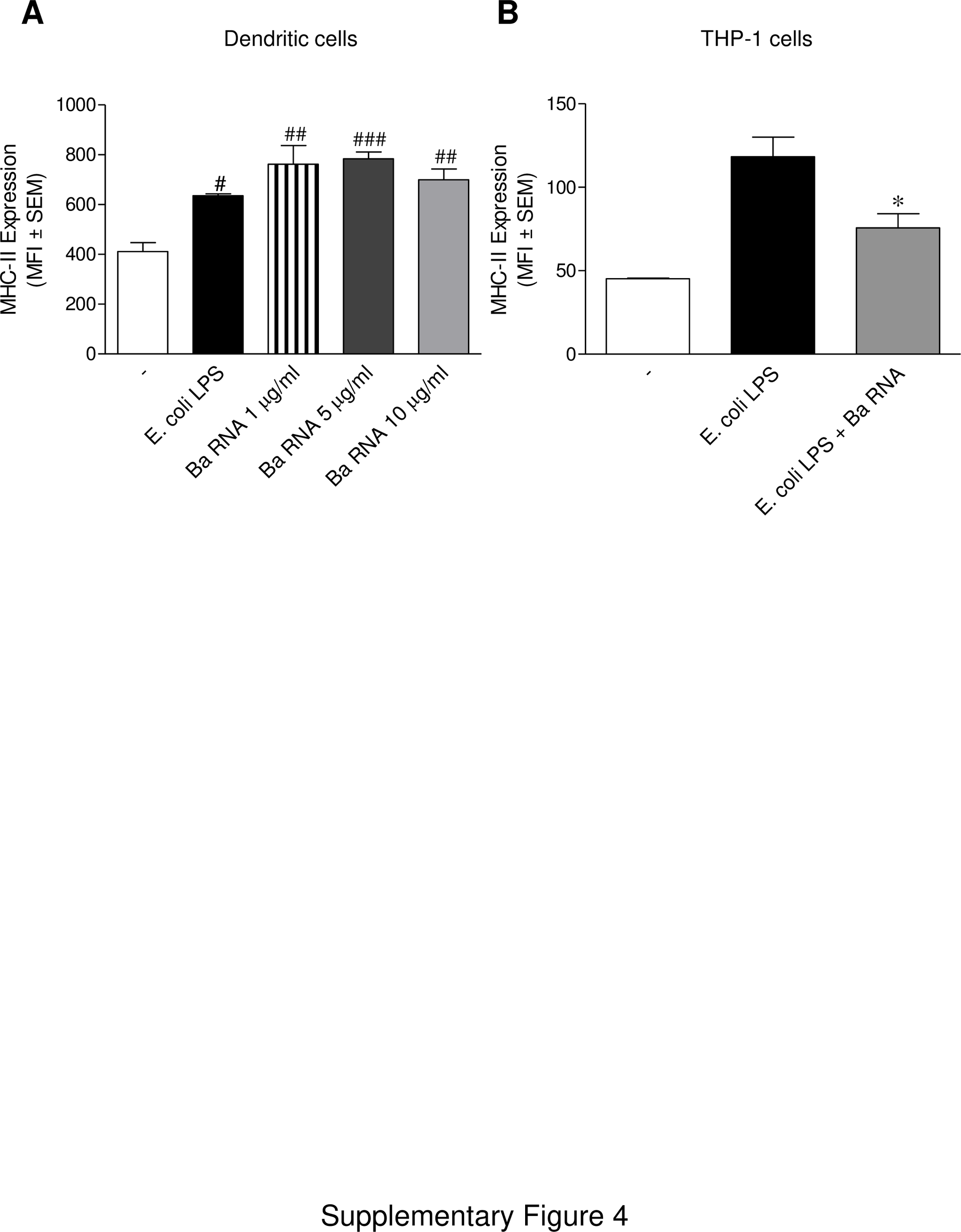

Supplement: Figure S4 — B. abortus RNA induced MHC-II expression on DCs while it inhibits the LPS-induced MHC-II on human monocytes. (A) DCs were treated with B. abortus RNA (1–10 μg/ml) or E. coli LPS (10 ng/ml) as a positive control of MHC-II induction for 24 h. (B) THP-1 cells were treated with B. abortus RNA (5 μg/ml) in the presence of E coli LPS (10 ng/ml) for 48 h. MHC-II expression was assessed by flow cytometry. Bars represent the arithmetic means ± SEM of three independent experiments. MFI, mean fluorescence intensity; #P < 0.05; ##P < 0.01; ###P < 0.001 vs. untreated cells; *P < 0.05 vs. LPS-treated cells. [file Image_4.TIF]

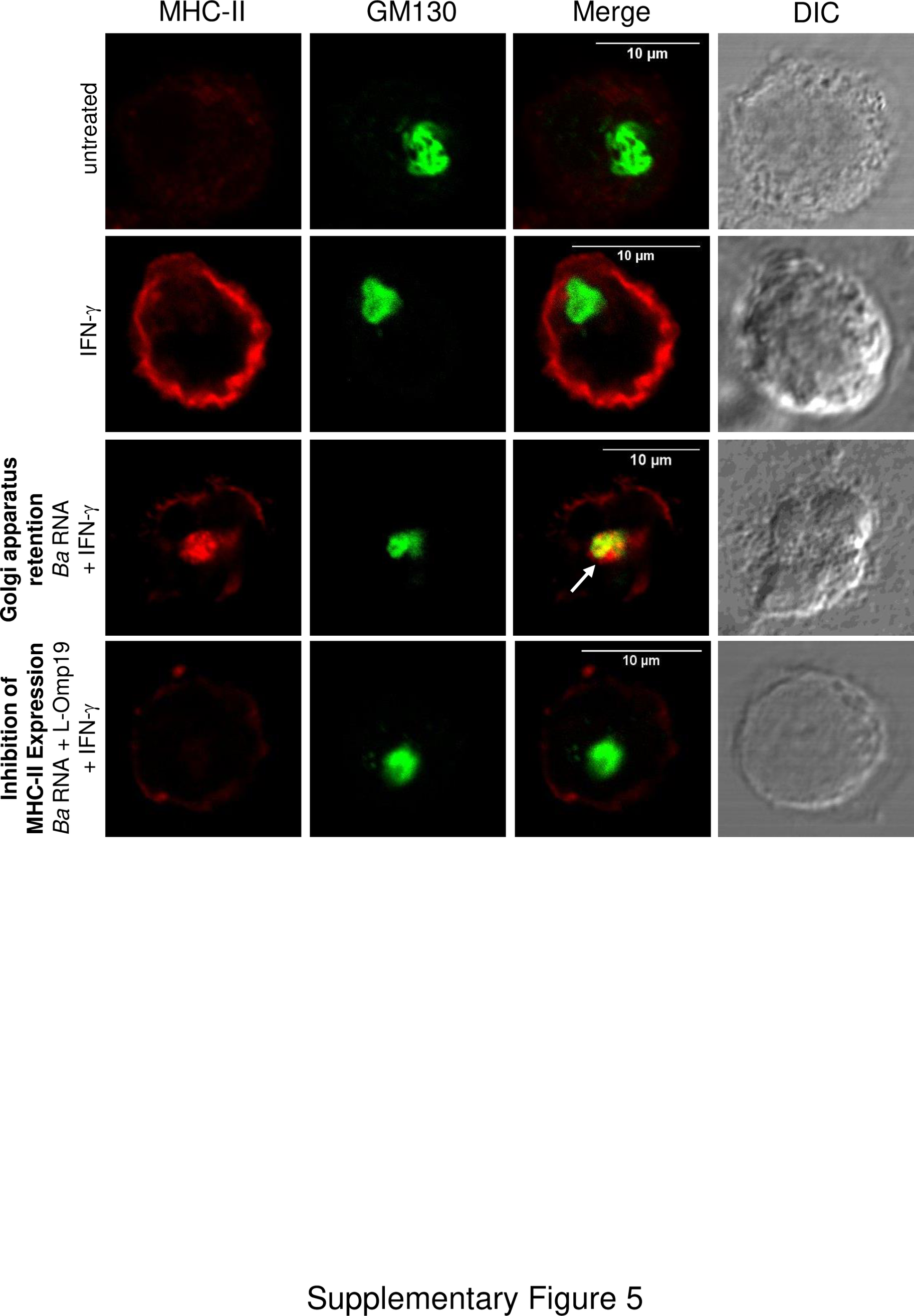

Supplement: Figure S5 — B. abortus RNA and lipoproteins down-modulate MHC-II mainly by MHC-II inhibition inside the cells. Zooms of confocal micrographs of THP-1 cells treated with B. abortus RNA (10 μg/ml) or B. abortus RNA (10 μg/ml) plus L-Omp19 (1 μg/ml) in the presence of IFN-γ, as representative figures of MHC-II down-modulation mechanisms (retention in Golgi apparatus and MHC-II inhibition). MHC-II was detected with a primary anti-human MHC-II Ab (L243) followed by Alexa 546-labeled secondary Ab (red). Golgi apparatus was detected using a mAb specific for GM130 followed by Alexa 488-labeled secondary Ab (green). DIC, differential interference contrast. [file Image_5.TIF]
